# Using postal questionnaires to evaluate physical activity and diet behaviour change: case study exploring implications of valid responder characteristics in interpreting intervention outcomes

**DOI:** 10.1186/1756-0500-7-725

**Published:** 2014-10-15

**Authors:** Judith A Cole, Paddy Gillespie, Susan M Smith, Molly Byrne, Andrew W Murphy, Margaret E Cupples

**Affiliations:** Department of General Practice and Primary Care, Queen’s University Belfast, Dunluce Health Centre, 1 Dunluce Avenue, Belfast, BT9 7HR UK; Economics, School of Business and Economics, NUI Galway, Galway, Ireland; Department of General Practice and HRB Centre for Primary Care Research, Royal College of Surgeons in Ireland, Lower Mercer Street, Dublin 2, Ireland; School of Psychology, Arts Millennium Building, NUI Galway, Galway, Ireland; Discipline of General Practice, Clinical Science Institute, NUI Galway, Galway, Ireland

**Keywords:** PROMs, Lifestyle, Self report questionnaires, Diet, Physical activity, Research methods

## Abstract

**Background:**

Patient reported outcome measures (PROMs) are used to evaluate lifestyle interventions but little is known about differences between patients returning valid and invalid responses, or of potential for bias in evaluations. We aimed to examine the characteristics of patients who returned valid responses to lifestyle questionnaires compared to those whose responses were invalid for evaluating lifestyle change.

**Methods:**

We conducted a secondary data analysis from the SPHERE Study, a trial of an intervention to improve outcomes for patients with coronary heart disease in primary care. Postal questionnaires were used to assess physical activity (Godin) and diet (DINE) among study participants at baseline and 18 month follow-up. Three binary response variables were generated for analysis: (1) valid Godin score; (2) valid DINE Fibre score; and (3) valid DINE Total Fat score. Multivariate analysis comprised generalised estimating equation regression to examine the association of patients’ characteristics with their return of valid responses at both timepoints.

**Results:**

Overall, 92.1% of participants (832/903) returned questionnaires at both baseline and 18 months. Relatively fewer valid Godin scores were returned by those who left school aged <15 years (36.5%) than aged 18 and over (50.5%), manual workers (39.5%) than non-manual (49.5%) and those with an elevated cholesterol (>5 mmol) (34.7%) than those with a lower cholesterol (44.4%) but multivariate analysis identified that only school leaving age (p = 0.047) was of statistical significance.

Relatively fewer valid DINE scores were returned by manual than non-manual workers (fibre: 80.8% v 86.8%; fat: 71.2% v 80.0%), smokers (fibre: 72.6% v 84.7%; fat: 67.5% v 76.9%), patients with diabetes (fibre: 75.9% v 82.9%; fat: 66.9% v 75.8%) and those with cholesterol >5 mmol (fat: 68.2% v 76.2%) but multivariate analysis showed statistical significance only for smoking (fibre: p = 0.013; fat: p = 0.045), diabetes (fibre: p = 0.039; fat: p = 0.047), and cholesterol (fat: p = 0.039).

**Conclusions:**

Our findings illustrate the importance of detailed reporting of research methods, with clear information about response rates, respondents and valid outcome data. Outcome measures which are relevant to a study population should be chosen carefully. The impact of methods of outcome measurement and valid response rates in evaluating healthcare requires further study.

## Background

A large body of evidence, from both clinical and observational studies, has been built up over recent decades to support the principle that a lifestyle which includes regular physical activity and a balanced, healthy diet helps to prevent disease [1]. The effectiveness of interventions incorporating different approaches to lifestyle change has been reported for both the primary and secondary prevention of coronary heart disease (CHD) [2, 3]. However, gaps remain in the evidence regarding optimal intervention design. Crucial to evaluating the effectiveness of lifestyle interventions are the methods chosen to assess changes in physical activity and diet.

Self-report measures of physical activity, such as questionnaires or diaries, are used widely although they are subject to recall bias [4, 5] and may have limited accuracy [6]. Objective measures such as accelerometers, pedometers or heart rate monitors [7] can also be used to assess physical activity but these are more expensive and study participants require instruction in their use. Similarly, a variety of measures can be used to assess diet, including specialist techniques involving doubly labelled water, direct observation, detailed food diaries and questionnaires: measurement errors can exist in every method [8]. Food frequency questionnaires are widely used, given that they are relatively easily administered, inexpensive and provide useful information [9] although multiple issues are relevant to their validity [10]. Higher numbers of items, giving greater detail, may increase validity [11] but higher response rates are more likely with shorter questionnaires [12].

The SPHERE Study (Secondary Prevention of Heart Disease in General Practice) [13, 14] aimed to evaluate individually tailored care plans to improve outcomes, including health-related behaviours, for patients with CHD in primary care. The study chose self-report measures to assess behaviour, using the Godin Leisure Time Questionnaire [15] to assess levels of physical activity and the Dietary Instrument for Nutrition Education (DINE) questionnaire [16] to assess diet.

The Godin questionnaire [15] provides a measure of type, duration and intensity of leisure-time exercise and is designed to be straightforward to use [17]. It has been validated [18] and tested for reliability [19] in studies in which subjects had opportunity to ask questions but other studies have reported posting it to participants [20, 21] without offering this facility. It is a four-item questionnaire, which gives examples of mild, moderate and strenuous exercise and asks how many times per week the respondent undertakes 15 minutes of each exercise category.

The DINE questionnaire [16] is a short questionnaire which is considered an acceptable alternative to more detailed diet recall questionnaires and food diaries. It gives a measure of dietary intake of fibre and fat, focusing on these because of their important association with cardiovascular disease and cancer and excluding other diet components in order to keep the questionnaire as short as possible. It was validated amongst workplace employees [16], to whom nurses administered the questionnaire: scores derived from it were compared with 4-day diet records. Face-to-face administration allowed ambiguous or unclear responses to be clarified immediately but in other studies DINE has been posted to participants [22, 23].

Within the SPHERE study, questionnaires were posted to participants and returned to practices by post, without opportunity for advice or review. Whilst the study achieved 92.8% follow-up [24], assessment of change in physical activity and in diet was possible only for, respectively, 38% and 75% of the total sample (these were the proportions of participants who provided valid responses at both time points). Thus, potential respondent bias may have implications for the interpretation of the evaluation. Whilst people who live in affluent areas are more likely to respond to questionnaires than those who live in deprived areas [12] differences in characteristics of patients with CHD who do and do not return valid responses to lifestyle questionnaires, administered by post, are not known.

This study aimed to examine possible respondent bias within the SPHERE Study by conducting a secondary data analysis to determine if characteristics of those who returned responses to postal questionnaires which allowed valid assessment of their physical activity and diet differed from those who did not. We also incorporated qualitative observations from a later follow-up study during which the researcher directly observed participants completing questionnaires.

## Methods

### Background: the SPHERE study

This study is set in the context of the SPHERE Study, a large cluster randomised trial conducted in the two different primary healthcare systems on the island of Ireland [13]. Briefly, participants were recruited from three different centres (Belfast, Northern Ireland (NI), UK; Galway, Republic of Ireland (RoI) and Dublin (RoI). Forty eight practices (16 from each centre) were randomly selected by an individual independent of the research team, using computer generated random numbers.

Lists of patients with known CHD were compiled by practice staff and those with significant mental or physical illness likely to impair capacity to participate in the intervention were excluded. Patients were invited, by post, from random order lists until 20 from each practice agreed to take part. They were posted questionnaires, including Godin and DINE, with reply paid envelopes which were posted back to the practice, for collection by the researchers, without review by practice staff. Six weeks were allowed for non-response, during which reminders were posted.

Baseline data collection was completed before randomisation into intervention and control groups to minimise potential recruitment bias. During the following 18 months (2005–2007) those in the intervention group attended their practice nurse or general practitioner for 4-monthly consultations at which lifestyle, risk factors and medication were reviewed. Goals for lifestyle change and targets were set, for review at each subsequent consultation. After 18 months, in addition to objective measurement of bio-physical risk factors, participants were again posted questionnaires following the same process as at baseline.

The SPHERE Study was granted ethical approval by the Irish College of General Practitioners and the Queen’s University research ethics committee.

### Qualitative study

A follow-up qualitative study of barriers and facilitators affecting lifestyle change was conducted after 4 years and has been reported previously [25]: 45 participants were interviewed and at the end of their interview they were invited to self-complete a Godin and a DINE questionnaire in the presence of the researcher who addressed any queries. Observational data relating to the completion of these questionnaires were extracted from that study in order to help contextualise the quantitative analyses described below.

Ethical approval for this follow-up study was granted by the Office for Research Ethics Committees (Northern Ireland), ref 10/NIR03/11 (April 2010), and the Irish College of General Practitioners Research Ethics Committee (May 2010).

### Data management

The aim of this current paper was to use data from the SPHERE Study to compare characteristics of participants who did or did not return questionnaires from which valid outcome measurements of physical activity and diet could be calculated at both baseline and follow-up.

An invalid response to the Godin questionnaire was considered to include any response other than a clear number of 15-minute PA sessions. Responses were scored to indicate the level of health benefit resulting from the reported exercise level. A score of <14 units indicates an insufficient level of activity with low health benefits; 14–23 indicates moderate activity with some health benefits; ≥24 indicates sufficient activity with substantial health benefits [17]. An invalid response to the DINE questionnaire was attributed to anything other than a stipulated answer [16]. Questions relate to the frequency of consumption of 19 food groups which contribute about 70% of the fat and fibre in a common Western diet. Each food group is given a score to reflect the nutrient content of a standard portion size, and scores are weighted according to frequency of consumption. No attempt was made to interpret unclear or missing responses. Within the current study, analysis was confined to DINE scores relating to fibre and total fat.

### Statistical analyses

Three binary response variables were generated for the statistical analysis: (1) valid Godin score; (2) valid DINE Fibre score; and (3) valid DINE Total Fat score. The statistical analyses examined rates of valid scores for each response variable for study participants with varying characteristics using a multivariate approach in which the choice of regression model was informed by the nature of the response variables under consideration and the hierarchical nature of the SPHERE dataset. In the case of the latter, there is a natural classification to the observations at the level of an individual general practice surgery, such that data are organised or clustered according to the practice with which a person is registered. With clustered data individual observations are not independent and multilevel analytical approaches are required for regression analysis [26]. In this study, generalised estimating equation (GEE) multivariate regression analysis was used to explore the effect of a range of independent variables on the three response variables of interest. When estimating a GEE model, in addition to identifying the appropriate linear predictor, it is also necessary to specify a suitable variance function, link function, and correlation structure [26]. The approach adopted in the current analysis was a binomial variance function and a log link function (both based on the binary nature of the response variables), and an exchangeable correlation structure (based on recommendations from the literature [26]). The patient characteristics which were included as independent variables in the multivariate regression models were: age, gender, years since diagnosis, history of myocardial infarction, systolic blood pressure, diastolic blood pressure, total cholesterol, smoking status, body mass index, SPHERE treatment allocation, age left school, marital status and occupational status.

The results of the multivariate analyses allow us to identify statistically significant associations for three response variables of interest, with statistical significance set at p < 0.05 for all analyses. The statistical analyses were conducted using the statistics package Stata 13.

## Results

In total, 903 patients took part in the SPHERE Study; all returned questionnaires at baseline and 832 (92.1%) completed response questionnaires at follow-up after 18 months.

### Response rates

Table 1 shows that the rates of return of valid Godin scores at both baseline and 18 month follow-up were similar (approximately 60%); a higher percentage returned valid DINE scores at baseline (95% fibre; 90% fat) than at follow-up (86% fibre; 81% fat).

**Table 1:** **Numbers who returned valid and invalid questionnaire scores at different time points during the SPHERE Study**

	Total questionnaires returned	
	Valid scores	Invalid scores
	N (%)	N (%)
Godin		
Baseline*	541 (59.1)	362 (40.9)
Follow-up**	523 (62.9)	309 (37.1)
DINE fibre		
Baseline*	861 (95.4)	42 (4.6)
Follow-up**	716 (86.1)	116 (13.9)
DINE total fat		
Baseline*	814 (90.1)	89 (9.90)
Follow-up**	673 (80.9)	159 (19.1)

*Baseline total N = 903; **Follow-up N = 832; 71 lost to follow-up due to: death; withdrawn consent; left practice; too ill; too busy; admitted to nursing home (44 intervention; 27 control).

Participants who did not return a follow-up questionnaire (n = 71) were excluded from our analyses. For the remaining 832 participants, characteristics were compared between those who returned questionnaires for which valid scores could be calculated at both time points and those who did not. Valid Godin scores were returned by 41.9% of the original sample (349/832), valid DINE fibre scores by 82.5% (686/832) and valid DINE total fat scores by 74.0% (616/832) at both time points.

### Descriptive comparisons of patient characteristics

In relation to the Godin questionnaire, fewer of the participants who left school at age 14 or under returned valid scores (36.5%), compared to those who left school aged 18 and over (50.5%) (Table 2); 39.1% of manual workers returned valid scores, whilst 49.8% of participants in non-manual occupations did so. Fewer of those whose total cholesterol was >5 mmol/l returned valid Godin scores (34.7%) than did those with lower cholesterol levels (44.4%).

**Table 2:** **Characteristics of patients at baseline and of those with valid and invalid responses to GODIN, DINE fibre and DINE fat questionnaires**

			GODIN		DINE fibre		DINE fat	
		Total study population (N = 832)*	Valid response	Invalid response	Valid response	Invalid response	Valid response	Invalid response
N (%)			349 (41.9)	483 (58.1)	679 (81.6)	153 (18.4)	616 (74.0)	216 (26.0)
Patient characteristics**			N (%)^‡^	N (%)^‡^	N (%)^‡^	N (%)^‡^	N (%)^‡^	N (%)^‡^
Age (years) (Mean; SD)		Mean 67.2; SD 9.53 (Range 29–91)	66.6 (9.77)	67.6 (9.33)	67.3 (9.57)	66.7 (9.37)	67.2 (9.58)	67.3 (9.41)
	70 years and older	340 (40.9%)	138 (40.6)	202 (59.4)	280 (82.4)	60 (17.6)	247 (72.6)	93 (27.4)
	<70 years	492 (59.1%)	211 (42.9)	281 (57.1)	399 (81.1)	93 (18.9)	369 (75.0)	123 (25.0)
Gender	Male	582 (70.0)	250 (42.9)	332 (57.1)	479 (82.3)	103 (17.7)	435 (74.7)	147 (25.3)
	Female	250 (30.0)	99 (39.6)	151 (60.4)	200 (80.0)	50 (20.0)	181 (72.4)	69 (27.6)
History of MI	Yes	415 (49.9)	182 (43.9)	233 (56.1)	338 (81.4)	77 (18.6)	310 (74.7)	105 (25.3)
	No	417 (50.1)	167 (40.0)	250 (60.0)	341 (81.8)	76 (18.2)	306 (73.4)	111 (26.6)
History of diabetes	Yes	145 (17.4))	58 (40.0)	87 (60.0)	110 (75.9)	35 (24.1)	97 (66.9)	48 (33.1)
	No	685 (82.3)	290 (42.3)	395 (57.7)	568 (82.9)	117 (17.1)	519 (75.8)	166 (24.2)
Elevated systolic BP***	Yes	281 (33.8)	115 (40.9)	166 (59.1)	229 (81.5)	52 (18.5)	202 (71.9)	79 (28.1)
	No	548 (65.9)	234 (42.7)	314 (57.3)	447 (81.6)	101 (18.4)	412 (75.2)	136 (24.8)
Elevated diastolic BP***	Yes	98 (11.8)	37 (37.8)	61 (62.2)	77 (78.6)	21 (21.4)	69 (70.4)	29 (29.6)
	No	733 (88.1)	312 (42.6)	421 (57.4)	601 (82.0)	132 (18.0)	546 (74.5)	187 (25.5)
Elevated cholesterol***	Yes	173 (20.8)	60 (34.7)	113 (65.3)	137 (79.2)	36 (20.8)	118 (68.2)	55 (31.8)
	No	621 (74.6)	276 (44.4)	345 (55.6)	514 (82.8)	107 (17.2)	473 (76.2)	148 (23.8)
Smoker	Smoker	117 (14.1)	48 (41.0)	69 (59.0)	85 (72.6)	32 (27.4)	79 (67.5)	38 (32.5)
	Non-smoker	685 (82.3)	296 (43.2)	389 (56.8)	580 (84.7)	105 (15.3)	527 (76.9)	158 (23.1)
BMI >25 kg/m2	Yes	673 (80.9)	278 (41.3)	395 (58.7)	551 (81.9)	122 (18.1)	493 (73.3)	180 (26.7)
	No	156 (18.6)	70 (44.9)	86 (55.1)	126 (80.8)	30 (19.2)	121 (77.6)	35 (22.4)
Study arm	Intervention	400 (48.1)	159 (39.8)	241 (60.3)	330 (82.5)	70 (17.5)	295 (73.8)	105 (26.3)
	Control	432 (51.9)	190 (44.0)	242 (56.0)	349 (80.8)	83 (19.2)	321 (74.3)	111 (25.7)
Marital status	Married/living with partner	565 (67.9)	250 (44.2)	315 (55.8)	471 (83.4)	94 (16.6)	427 (75.6)	138 (24.4)
	Single/widowed/separated/divorced	241 (28.9)	92 (38.2)	149 (61.8)	200 (82.9)	41 (17.1)	180 (74.7)	61 (25.3)
Age left school (years)	14 and under	337 (40.5)	123 (36.5)	214 (63.5)	280 (83.1)	57 (16.9)	244 (72.4)	93 (27.6)
	15 to 17	335 (40.3)	147 (43.9)	188 (56.1)	279 (83.3)	56 (16.7)	257 (76.7)	78 (23.3)
	18 and over	95 (11.4)	48 (50.5)	47 (49.5)	82 (86.3)	13 (13.7)	78 (82.1)	17 (17.9)
Occupation	Manual	281 (33.8)	110 (39.1)	171 (60.9)	227 (80.8)	54 (19.2)	200 (71.2)	81 (28.8)
	Non manual	325 (39.1)	162 (49.8)	163 (50.2)	282 (86.8)	43 (13.2)	260 (80.0)	65 (20.0)

*Total N = 832; numbers for some characteristics are less due to incomplete data; ^**‡**^percentages refer to row totals.

**Baseline data; ***Elevated SBP defined as systolic blood pressure >140 mmHg; elevated DBP defined as diastolic blood pressure >90 mmHg; elevated cholesterol defined as serum cholesterol >5.0 mmol/l.

Rates of return of valid scores observed for participants of different gender, age, marital status, study arm, BMI, blood pressure, history of MI or diabetes, or smoking status were similar.

In relation to the DINE fibre questionnaire, a smaller proportion of participants in manual occupations (80.8%) than in non-manual occupations (86.8%) returned valid scores. There was a trend towards those who left school at an older age being more likely to return valid scores than those who left school earlier. More of those who reported being non-smokers returned valid fibre scores than smokers (84.7% v 72.6%), Fewer of those without a history of diabetes than of those with diabetes returned valid responses (82.9% v 75.9%). Rates of valid and invalid DINE total fat scores were similar to those for DINE fibre in relation to history of diabetes, total cholesterol, smoking status and occupation (Table 2).

### Multivariate analysis of patient characteristics

Table 3 presents the results from the multivariate regression analyses. In the Godin score analysis, after controlling across the full set of patient characteristics, only a school leaving age of 18 or over was found to be statistically significant. The regression coefficient indicates a positive and statistically significant response effect (0.508; p = 0.047). In the DINE fibre analysis, a history of diabetes (−0.506; p = 0.039) and smoking (−0.682; p = 0.013) were both associated with negative and statistically significant response effects. Similarly, in the DINE total fat score analysis, a history of diabetes (−0.457; p = 0.047), smoking (−0.529; p = 0.045), and high total cholesterol (−0.455; p = 0.039) were associated with negative and statistically significant response effects.

**Table 3:** **Multivariate analysis of characteristics of patients with and without valid GODIN, DINE fibre and DINE fat scores at baseline**

Instrument		GODIN		DINE fibre		DINE total fat	
Patient characteristics**		Coefficient	p-value	Coefficient	p-value	Coefficient	p-value
Age		−0.010	0.238	−0.01	0.695	−0.006	0.616
Gender	Male	−0.044	0.801	0.174	0.440	−0.070	0.744
	Female	Ref*		Ref		Ref	
History of MI	Yes	0.040	0.790	−0.258	0.198	−0.235	0.208
	No	Ref		Ref		Ref	
History of diabetes	Yes	−0.037	0.854	−0.506	**0.039**	−0.457	**0.047**
	No	Ref		Ref		Ref	
Elevated systolic BP***	Yes	0.074	0.680	0.082	0.735	0.009	0.966
	No	Ref		Ref		Ref	
Elevated diastolic BP***	Yes	−0.318	0.218	−0.379	0.238	−0.330	0.267
	No	Ref		Ref		Ref	
Elevated cholesterol***	Yes	−0.283	0.140	−0.324	0.178	−0.455	**0.039**
	No	Ref		Ref		Ref	
Smoker	Smoker	−0.109	0.636	−0.682	**0.013**	−0.529	**0.045**
	Non-smoker	Ref		Ref		Ref	
BMI >25 kg/m2	Yes	−0.239	0.222	−0.005	0.983	−0.225	0.368
	No	Ref		Ref		Ref	
Study arm	Intervention	−0.159	0.293	0.141	0.488	−0.023	0.909
	Control	Ref		Ref		Ref	
Marital status	Married/living with partner	0.233	0.204	−0.228	0.366	0.025	0.911
	Single/widowed/separated/divorced	Ref		Ref		Ref	
Age left school (years)	14 and under	Ref		Ref		Ref	
	15 to 17	0.240	0.147	−0.004	0.987	0.024	0.905
	18 and over	0.508	**0.047**	0.134	0.712	0.475	0.179
Occupation	Manual	Ref		Ref		Ref	
	Non manual	0.226	0.243	0.306	0.267	0.275	0.250

*Total N = 832; numbers for some characteristics are less due to incomplete data; Ref refers to reference category for valid responses for each characteristic.

**Baseline data; ***Elevated SBP defined as systolic blood pressure >140 mmHg; elevated DBP defined as diastolic blood pressure >90 mmHg; elevated cholesterol defined as serum cholesterol >5.0 mmol/l.

Figures in bold are statistically significant.

*Qualitative study data* Of the 45 interviewees 2 declined to complete either a Godin or a DINE questionnaire due to time constraints. Of the remainder, 72.1% (31/43) had valid scores for the Godin questionnaire; 93.0% (40/43) had valid scores for DINE fibre; scores for total fat were not computed. Questions asked by participants related to advice as to whether certain activities could be counted as ‘exercise’, help in translating activities into 15 minute periods of different intensities of exercise, whether certain foods ‘fitted’ the categories presented in DINE and translation of their diet recall into numbers of servings per week. It was observed that some participants did not take time to read questions carefully or take account of different question construction requiring a different format of answer.

## Discussion

### Summary of main findings

These findings show that study participants with a lower level of education returned relatively fewer valid Godin scores for assessment of change in their self-reported physical activity. Relatively fewer valid DINE scores were returned by smokers, people with diabetes and total cholesterol >5 mmol/l.

The Godin questionnaire seemed to be challenging for respondents, all of whom had CHD: more than 50% returned questionnaires which did not allow a calculation of change of their level of physical activity. The DINE seemed to be more straightforward to answer, with almost all at baseline and 80% at follow-up giving responses which allowed calculation of change in their fibre intake.

#### Comparison with existing literature

Details of the methods of administration, response rates and valid completion rates of lifestyle behaviour questionnaires have often not been reported clearly in previous studies of behaviour change interventions. For example, Hunt-Shanks et al. [20] investigated the effect of exercise among cardiac patients by posting Godin questionnaires, but response rates were unclear. Trinh et al. [21] examined correlates of physical activity among kidney cancer survivors by posting a survey ‘package’, including a Godin questionnaire. Completed questionnaires were returned by 42.5% (703/1,654) which is similar to our findings of the rate of valid returns: they found no differences in age, sex or surgery rate between responders and non-responders but, in contrast to our report, they did not report if any incomplete or invalid responses were received.

Rosenburg et al. [27] used postal Godin questionnaires among a disabled population and commented that the examples of exercise were inappropriate but did not report clearly details of responses received. A reason for poor return of valid scores in our study may be that participants considered that the questions were not relevant to them because of impaired mobility. Evaluation of intervention effectiveness is only meaningful if the outcome measures are appropriate to the study population which, in secondary intervention studies, often includes older and multimorbid people, with cognitive or physical impairments. Involvement of patients from the target study population in all stages of research design planning and in interpreting findings should help to ensure that relevant outcome measures are chosen and measured appropriately.

Our finding that return of valid scores is associated with higher educational attainment is in keeping with findings of Cash et al. [28] who surveyed diet and physical activity behaviours among employees who were mostly high school graduates or higher. Godin scores were reported for approximately 80% of the sample but reasons for the full sample not being included are not given or discussed.

A previous study [6] which used a self administered Godin questionnaire, sent and returned by post, in testing the effectiveness of an intervention for patients with multiple sclerosis offered a $10 incentive for a completed return. The report suggests that 90.5% (19/21) completed questionnaires successfully. Thus, the offer of an incentive may boost response rates but there needs to be recognition that an incentive which is more attractive to some participants than others may also introduce a further source of response bias in evaluating an intervention.

In keeping with our findings, good response rates have been reported for DINE in previous studies. John et al. [23] mailed a DINE questionnaire to participants in a study of a healthy eating intervention before they attended appointments with a nurse. Whilst a 95% (655/690) response rate was reported, it is unclear if the DINE was reviewed during the appointment and it was noted that participants were of a higher socioeconomic class than the UK average. Steptoe et al. [29] also used DINE to examine the effect of eating behaviour counselling but their report did not state if patients completed this in the presence of a researcher or health professional from whom they could seek advice. At 12-month review it was completed by 80% of the original participants (218/271) but no comments were made regarding invalid responses. Further detail of methods of administration and completion of outcomes in these studies would be helpful.

#### Strengths and limitations

The SPHERE Study involved a large sample (n = 903) with opportunity to compare participants’ responses to self report questionnaires at baseline and follow-up time points. The completeness of follow-up (98.2%) allowed comparisons between those who did and did not return valid scores in relation to many different characteristics.

Administering the questionnaires by post without giving participants an opportunity to ask advice if they had uncertainty regarding any questions may be considered to be a weakness of the original study, which limited the possibility of maximising the number of valid responses. Analysing returned questionnaires, however, without making assumptions regarding responses which were not in strict accordance with instructions reflects accurately the way in which the questions were answered without assistance and without the possible influence of observer bias.

A further strength of this study is its integration of quantitative and qualitative data. The researchers’ observations of the process of questionnaire completion confirmed how study participants struggled to understand the content and construct of some questions and to respond appropriately. Some had difficulty following the format of the forms, illustrating how completion of questionnaires requires literacy and an ability to follow instructions correctly [30] and the relevance of our findings which associate higher educational status with the return of valid responses.

#### Implications

Our results suggest that research study participants may have difficulty in providing appropriate responses to the Godin and DINE questionnaires when these are posted and completed without access to support, resulting in a lower valid response rate than when administered face-to-face. Having someone available to give advice is not always practical because of time, travel and financial constraints and whilst it may increase the number of meaningful responses, completeness of evaluation and confidence in conclusions regarding intervention effectiveness, it may also introduce bias due to the influence of the source of advice. However, self-completion of questionnaires, with review by a health professional or researcher and revision of responses if indicated, may reflect a pragmatic approach which is relevant to real-world clinical settings. For example, some respondents may leave boxes blank when an appropriate response would be to record a zero, indicating no relevant food consumption or activity. Careful consideration may be given to the possibility of making assumptions to interpret some responses in postal questionnaires, to allow these to be included in analyses.

The importance of reporting information about valid response rates is indicated by our findings that those with higher levels of cardiovascular risk, associated with manual occupational status and lower educational attainment, with higher cholesterol levels and with diabetes were more likely to return invalid responses. Those who are aware of their higher level of risk may have feelings of guilt about their eating and physical activity behaviours which may influence valid completion of questionnaires. However, exclusion of such individuals from analysis of the outcomes of lifestyle change interventions has implications for conclusions regarding their overall effectiveness.

Our findings also illustrate the importance of ensuring that outcome measures used in evaluating interventions are appropriate for the target population. There is an increasing focus on developing health services that are patient centred [31] and patient reported outcome measures (PROMs) can be used to achieve this aim [32]. However, whilst these can be useful in monitoring the quality of care delivery [33], potentially harmful results may arise from inappropriate methodology, relating to the design of measures chosen or the method of their administration and data collection, leading to evaluations which are applicable only to a skewed sample of the eligible population. Attention should be paid to patients’ literacy [30] and health literacy [34]. The readability of information and patients’ views are of paramount importance in developing management plans [35] and should be key determinants of the choice and design of appropriate PROMs, such as lifestyle questionnaires.

## Conclusion

More detailed reporting of research methods and findings, with clear information about response and valid completion rates is important to inform the evaluation of interventions to change diet and physical activity behaviour. Close attention should be paid to the setting and context of studies which have been used to validate outcome measures chosen to evaluate healthcare interventions. Further research is required to determine which lifestyle questionnaires are most suitable and how they should be administered in order to maximise valid outcome data, particularly for populations such as older adults with chronic illness and those with socio-economic disadvantage or lower levels of education. The impact of methods of outcome measure administration and valid response rates in evaluating healthcare requires further study.
